# The effect of an exercise intervention on adolescent idiopathic scoliosis: a network meta-analysis

**DOI:** 10.1186/s13018-023-04137-1

**Published:** 2023-09-04

**Authors:** Yonghuan Chen, Zhendong Zhang, Qiuhan Zhu

**Affiliations:** 1 Department of Physical Education, Pukyong National University, Busan, South Korea; 2 School of Physical Education, Zhengzhou University, Zhengzhou, China; 3 School of Physical Education, Zhengzhou Sias University, Zhengzhou, Henan China

**Keywords:** Exercise intervention, Adolescent, Cobb's angle, Scoliosis, Network meta-analysis

## Abstract

**Purpose:**

To explore the effect of exercise intervention on adolescent idiopathic scoliosis (AIS), various exercise forms were compared and the sequence of the possibility of improving the effect of each exercise form was sorted out. We expect that our findings will provide clinicians and patients with more effective treatments and references.

**Method:**

A thorough search was done on CNKI, Wanfang, WOS, Cochrane library, Embase, PubMed, Scopus and obtained the publication time from the database establishment to May 6, 2023. The relevant contents of the literature that passed the screening criteria were extracted, including relevant information about the sample, first author, intervention measures, intervention time, and outcome indicators. Analysis was performed by Review Manager 5.4 and Stata17.0.

**Result:**

The study finally included 12 articles with 538 samples. After comparison, it was found that exercise interventions to reduce Cobb's angle were more effective than conventional therapies and reached a statistically significant difference. Compared with conventional therapy, core strength training, Physiotherapeutic Scoliosis-Specific Exercise (PSSE), yoga, Schroth, and sling reduced the Cobb angle by an average of 3.82 degrees, 3.79 degrees, 4.60 degrees, 3.63 degrees, and 3.30 degrees, respectively. However, the therapeutic effects on AIS did not show statistically significant differences between the exercise interventions. According to the SUCRA value and the cumulative probability, the MeanRank of improving the AIS effect by various sports intervention measures as follows: yoga (2.2), core strength training (2.8), PSSE (2.8), Schroth exercise (3.2), and sling exercise (4.0).

**Conclusion:**

Exercise intervention can significantly improve AIS. There was no significant difference in the improvement effect of AIS among different exercise forms. Yoga may have the best effect on AIS improvement.

## Introduction

As a very important physiological structure of the human body, the spine plays an important role in cushioning vibration, supporting the trunk, and supporting movement. However, the pathological changes of the spine caused by genetic [1, 2] and environmental [3] have become a common "civilization disease." Generally speaking, spinal disease refers to the lesions caused by congenital, traumatic, degenerative, and other reasons, resulting in damage to the spine itself, intervertebral discs, bones, spinal cord, nerve roots, and muscles and ligaments around the spine. Clinically, scoliosis can be divided into congenital or developmental scoliosis and deformity: spinal stenosis; spinal instability; spinal cord or nerve root injury; spinal infection; vertebral osteoporosis; spinal-related soft tissue diseases; degenerative disorders of the spine; spondylolisthesis; spinal trauma; spinal tumor; spinal rheumatism, rheumatoid diseases, ankylosing spondylitis. There are 14 types of spine-related diseases, caudal and sacral vertebral diseases [4]. Idiopathic scoliosis belongs to the first spinal disease according to clinical classification. The International Association for the study of Scoliosis describes the diagnostic criteria for scoliosis as the patient is in a standing position and the Cobb angle of the spine is greater than 10 degrees [5].

According to the time of occurrence of the lesion, it can be divided into congenital [6], acquired, and AIS, and acquired scoliosis can be divided into neuropathic and chest pathological scoliosis according to the cause of the lesion [7]. Idiopathic scoliosis disease rate is also the most common, the highest incidence of one, and because this type of scoliosis often occurs in the adolescent stage, so it is also called adolescent idiopathic scoliosis (AIS) [8]. At present, the pathogenesis of AIS has not been fully identified, and the mainstream viewpoints of the academic community include genetic factors [9, 10], skeletal muscle system and biomechanical factors [11], biochemical factors [12, 13], nervous system factors [14], and growth and development factors [15]. According to epidemiological studies, the prevalence of adolescent idiopathic scoliosis in the world is approximately 0.93–12% [16]. AIS not only has adverse effects on the body shape and quality of life of patients [17], but also has certain effects on the cardiopulmonary functions of patients [17, 18] and even causes organ failure and paralysis of patients [19]. At the same time, studies by scholars have shown that AIS is also harmful to the mental health of patients, and most of the AIS patients suffer from mental health problems such as anxiety, depression, and fear [20]. Therefore, the diagnosis and active treatment of AIS is of great significance. For AIS rehabilitation means, there are surgical and non-surgical treatments. Non-surgical treatment includes conventional therapy (education, brace treatment, and nursing), electrical stimulation therapy [21], exercise therapy, traditional Chinese bone-setting massage [22, 23], and other treatment methods. The findings of scholars have shown that all these treatment modalities play a role in the rehabilitation of AIS patients to varying degrees. Conventional therapies play a positive role in the rehabilitation of AIS patients and are widely used in clinical practice, but scholarly studies have shown that conventional therapies such as bracing can reduce patients' physical self-esteem and trigger psychological anxiety [24]. Kotwicki [25] found that AIS patients were psychologically more sensitive to wearing braces compared to scoliosis. Therefore, conventional therapy was chosen as a control group. Meanwhile, the role played by exercise interventions in the treatment of scoliosis is becoming more and more important. Exercise intervention measures for AIS include yoga [26, 27], pilates [28], sling exercise [29], PSSE [30–32], core strength training [33–37], massage [39], acupuncture [40], etc. However, there is no empirical comparative study on which of these interventions have the most significant effect. Therefore, this study used mesh meta-analysis to indirectly compare the intervention effects of various exercise forms on AIS and ranked the intervention effects of each exercise form, in order to provide reference and basis for medical workers and patients when choosing more effective exercise prescriptions.

## Materials and methods

The study is based on a systematic review of the research on healthcare interventions and the evaluation statement of meta-analysis (PRISMA) [41], strictly controls the meta-analysis process, and establishes the screening criteria of the literature according to the principles of PICOS.

*Inclusion criteria*: 1. The subjects (P) were patients with adolescent idiopathic scoliosis (Cobb angle greater than 10 degrees). 2. Intervention measure (I) is various forms of exercise; the exercise interventions covered in this article include core strength training, Schroth exercise, sling exercise, yoga, and PSSE; the control measures (C) are conventional treatments, including education and awareness, brace therapy, and daily care. 3. The outcome index (O) is Cobb angle (the angle of the most inclined vertebrae at both ends of the spine); 4. the study design (S) was a randomized controlled trial (RCT).

*Exclusion criteria*: 1. The same literature collected in each database; 2. conference articles, review research, and secondary research literature; 3. in addition to exercise intervention and routine intervention, there are any other forms of intervention;4. the valid data cannot be obtained, or the experimental results are not reported by Cobb angle.

### Literature retrieval strategy

Idiopathic scoliosis, sport/exercise, taichi, yoga, pilates, sling exercise, PSSE, and Schroth, were searched in CNKI, Wanfang, WOS, Cochrane library, Embase, Scopus, PubMed, and other databases. The search deadline is May 6, 2023.

### Literature screening and data extraction

Each of the two researchers made a preliminary screening of the collected literature and extracted the content and information of the article, and then the two researcher cross-checked. If the verification results of the two researcher are inconsistent, seek the third researcher to discuss and decide together, and if the results are agreed, they will be included in the study. The extracted information includes 1. The name of the first author, the time of publication, and the nationality of the author; 2. sample size, sample age. 3. Intervention measures, duration of intervention. 4. Outcome indicators and experimental results.

### Cochrane risk of bias assessment

**Fig. 1:**
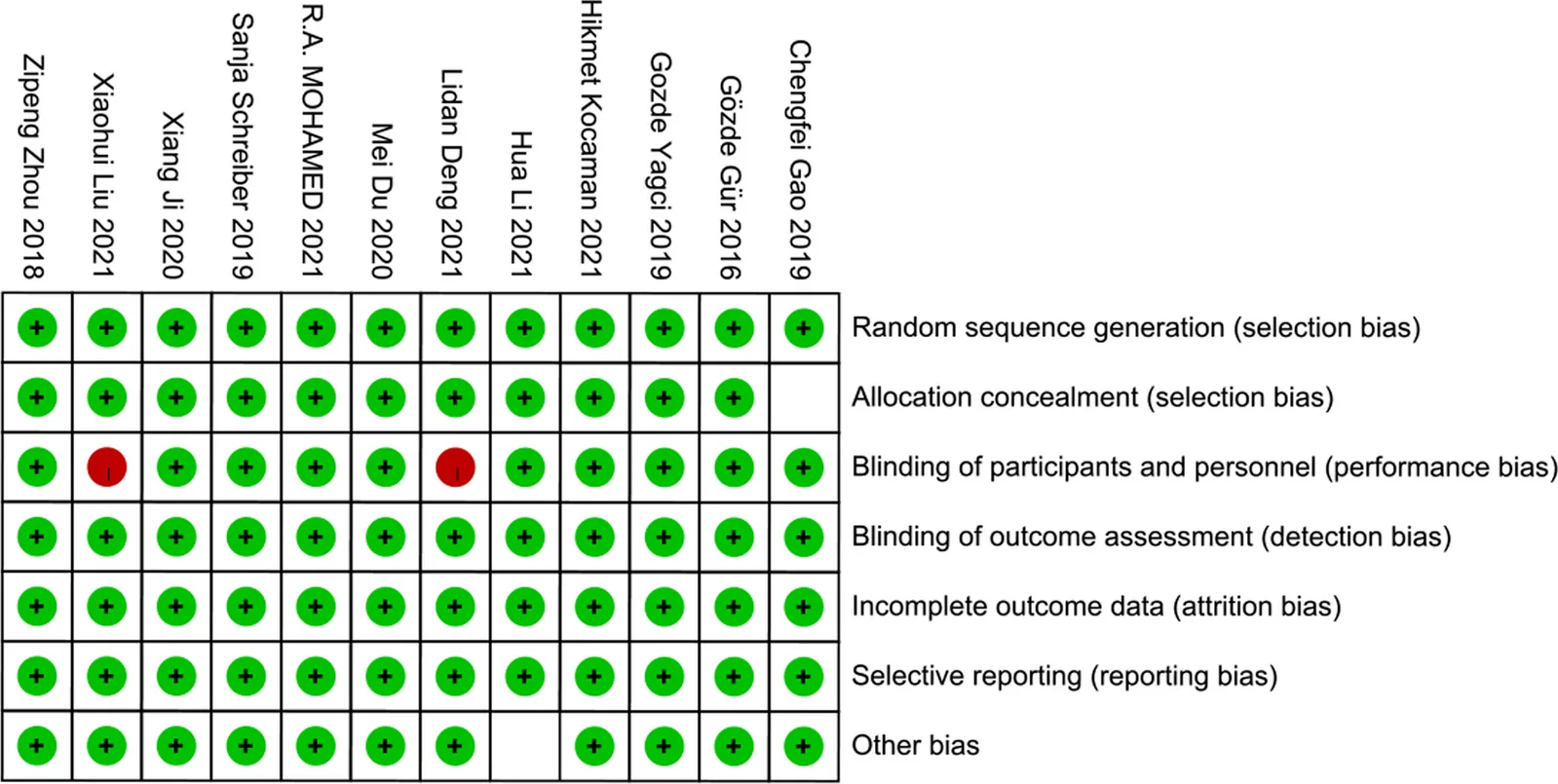
Risk of bias summary

The study was carried out strictly according to bias risk assessment tools and related norms. The evaluation included seven aspects: random process bias, intervention deviation bias (distribution effect and compliance effect), missing outcome data bias, outcome measurement bias, selective reporting bias, and other biases. If all levels show low risk, the overall bias risk is judged to be "low"; if some levels show "certain risk" but no "high risk," the overall bias risk is determined to be "medium"; if only one level is "high risk," the overall bias risk is determined to be "high" [42]. The risk assessment results are shown in Fig. 1, Xiaohui Liu and Lidan Deng were judged to be high risk on perform bias, Chengfei Gao was judged to be at unclear risk on selection bias and Hua Li on other bias.

### Statistical analysis

ReviewManager5.4 and Stata17.0 software were used to analyze the data. The final result is explained according to the combined effect and 95% CI. The research uses the Cochrane risk assessment tool to evaluate the literature. The publication bias of the literature was judged by the Egger test. In the analysis of reticular Meta, if there is a closed loop in each form of exercise intervention, the global and local inconsistencies are tested. The possibility of each exercise form interfering with the effect of AIS was ranked by SUCRA value and optimal probability.

## Result

### Basic features of the included literature

**Fig. 2:**
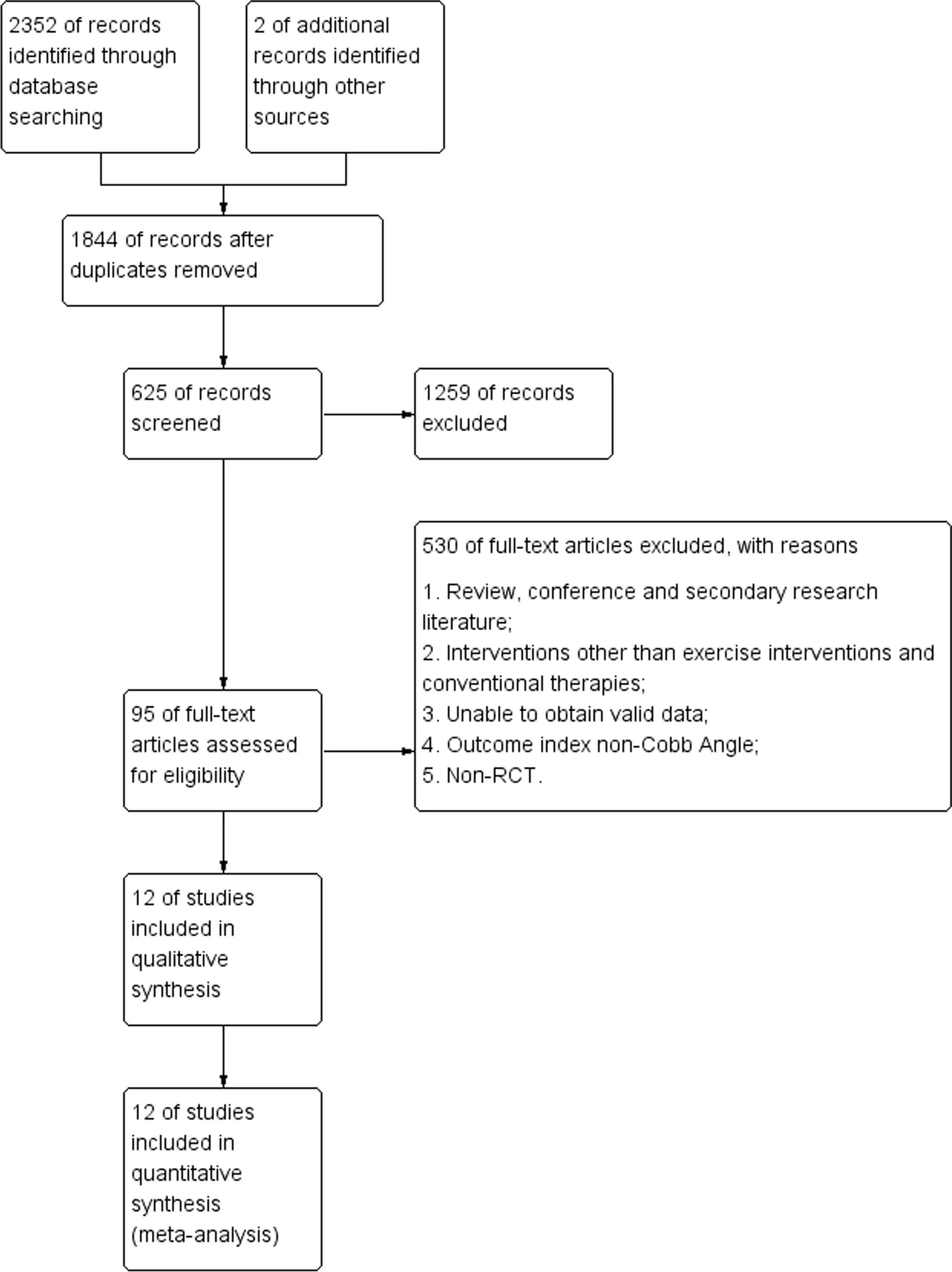
Flowchart of eligible study selection

As shown in Fig. 2 and Table 1. A total of 2352 relevant literature were obtained, and 12 valid literature was included after screening according to the above criteria. The study subjects were all patients with adolescent idiopathic scoliosis, and the total sample size was 538, among which the sample size of the experimental group and the control group was the same, and both were 269. Among the 12 articles, 6 articles were comparative studies of core strength training and conventional therapy [32–37], 2 articles were comparative studies of PSSE and conventional therapy [30, 31], 2 articles used yoga as an intervention [26, 27], and 1 article each used Schroth exercise [38] and sling exercise [29] as an intervention.

**Table 1:** Basic features of the included literature

First author and year of publication	Sample size T/C	Gender M/F	Age T/C	Intervention T/C	Intervention duration/week	Outcome measures
Xiang Ji (2020)	13/13	8/18	10.09 ± 1.92/10.69 ± 2.01	Core strength training/Conventional therapy	12	Cobb angle
Gözde Gür (2016)	13/13	1/25	14.2 ± 1.8/14 ± 1.6	Core strength training/Conventional therapy	10	Cobb angle
Hikmet Kocaman (2021)	14/14	7/21	14.07 ± 2.37/14.21 ± 2.19	Core strength training、Schroth exercise/Conventional therapy	10	Cobb angle
Sanja Schreiber (2019)	25/25	3/47	13.5 ± 0.7/13.3 ± 0.6	PSSE/Conventional therapy	24	Cobb angle
Mei Du (2020)	8/8	16/0	18.34 ± 0.89/18.91 ± 0.93	Yoga/Conventional therapy	12	Cobb angle
Xiaohui Liu (2021)	8/8	16/0	18.34 ± 0.89/18.91 ± 0.93	Yoga/Conventional therapy	12	Cobb angle
Gozde Yagci (2019)	15/15	0/30	14 ± 1.3/14.2 ± 1.5	Core strength training/Conventional therapy	16	Cobb angle
Chengfei Gao (2019)	23/22	9/36	12.22 ± 1.35/12.14 ± 1.32	PSSE/Conventional therapy	24	Cobb angle
Mohamed (2021)	17/17	0/34	14.50 ± 1.20/14.90 ± 1.40	Schroth exercise/Conventional therapy	24	Cobb angle
Hua Li (2021)	50/50	42/58	34.52 ± 5.68/33.86 ± 5.71	Sling exercise/Conventional therapy	4	Cobb angle
Lidan Deng (2021)	49/49	49/49	10.13 ± 2.29/10.16 ± 2.30	Core strength training/Conventional therapy	12	Cobb angle
Zipeng Zhou (2018)	40/40	33/47	24.7 ± 4.8/24.6 ± 4.6	Core strength training PSSE/Conventional therapy	12	Cobb angle

### Heterogeneity assessment and publication bias

After extracting the experimental results of the 12 literature included, the data effect size before and after the experiment was combined to obtain the change of the data before and after the exercise intervention. The heterogeneity among studies was found to be low (*I*^*2*^ = 37.7%, *P* < 0.090). In terms of publication bias of the literature, the Egger test is applied, and the result shows *P* = 0.013, indicating that there is significant publication bias.

### Network evidence map and consistency check

**Fig. 3:**
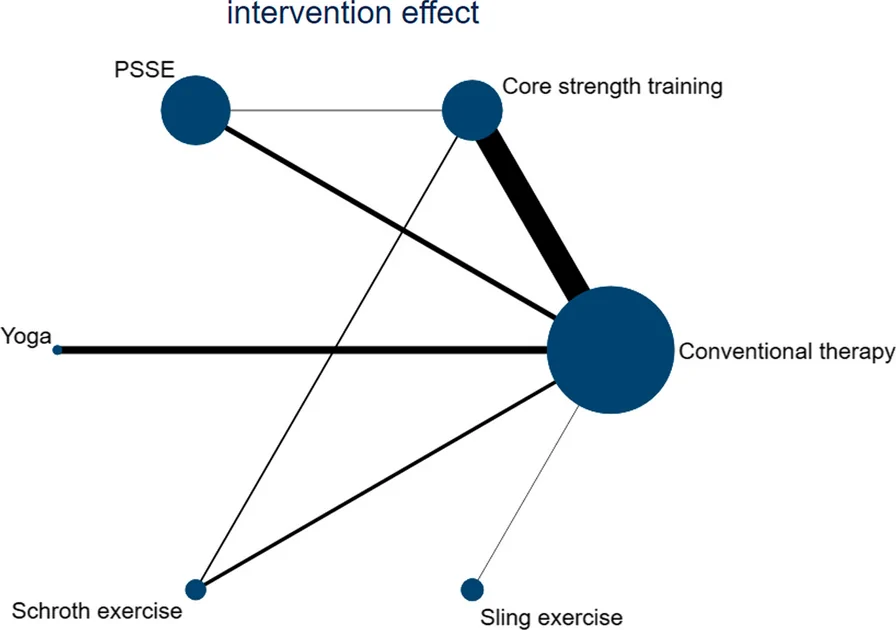
Network evidence for each intervention. Each node represents an intervention. The size of the nodes was positively correlated with the number of times the intervention was used in the experiment. The width of the lines was positively correlated with the number of experiments comparing the two interventions

Before analysis, it is important to understand the relationship between studies. [43].A network of evidence maps can show which interventions were compared directly in the experiment and which were compared indirectly. The thickness of the lines in the center represents the number of studies, and the thicker the lines are, the more such interventions are used. Figure 3 shows the thickest line between core strength training and conventional therapy, indicating the largest number of direct comparisons between the two studies. A closed loop is formed between core strength training (B), PSSE (C), and conventional therapy (A), and core strength training (B), Schroth exercise (E), and conventional therapy (A). Then, the global inconsistency test (*P* = 0.7824), the loop inconsistency test [*P* = 0.152 for loop A-B-C; *P* = 0.792 for ring A-B-E], and the node splitting method were used to test the local inconsistency, and the results showed that *P* was all greater than 0.05, indicating that the studies were consistent. Therefore, the consistency model is finally adopted for analysis.

### Relative effect estimation

Network meta-analysis can directly compare the effects of each intervention, as well as compare the relative effects of any pair of interventions. Among the 5 interventions selected in this paper, there are 15 comparative results of relative effects. These results are presented in the form of league tables. As shown in Table 2, this table shows the results of direct and indirect comparisons of all interventions. The remaining interventions significantly reduced Cobb angle relative to conventional therapies. Core strength training reduced the Cobb angle by 3.82 degrees on average (95% CI: 2.90, 4.74). The PSSE reduced the Cobb angle by 3.79 degrees on average (95% Ci: 3.07, 4.51). Yoga reduced the Cobb angle by an average of 4.60 degrees (95% CI: 1.11, 8.08). Schroth reduced the Cobb angle by an average of 3.63 degrees (95% CI: 2.19, 5.06). Sling exercise reduced Cobb angle by 3.30 degrees on average (95% CI: 2.41, 4.19). By looking at the first column of this table, we can see that all interventions have a significant reduction effect on Cobb angle and are more effective than conventional therapies.

**Table 2:** Comparison of intervention effects of each intervention measure

Conventional therapy	− 3.82 ( − 4.74, − 2.90)	− 3.79 ( − 4.51, − 3.07)	− 4.60 ( − 8.08, − 1.11)	− 3.63 ( − 5.06, − 2.19)	− 3.30 ( − 4.19, − 2.41)
3.82 (2.90,4.74)	Core strength training	0.03 ( − 1.02,1.08)	− 0.78 ( − 4.38,2.83)	0.19 ( − 1.38,1.77)	0.52 ( − 0.76,1.81)
3.79 (3.07,4.51)	− 0.03 ( − 1.08,1.02)	PSSE	− 0.81 ( − 4.37,2.75)	0.16 ( − 1.42,1.75)	0.49 ( − 0.66,1.64)
4.60 (1.11,8.08)	0.78 ( − 2.83,4.38)	0.81 ( − 2.75,4.37)	Yoga	0.97 ( − 2.80,4.74)	1.30 ( − 2.30,4.90)
3.63 (2.19,5.06)	− 0.19 ( − 1.77,1.38)	− 0.16 ( − 1.75,1.42)	− 0.97 ( − 4.74,2.80)	Schroth exercise	0.33 ( − 1.36,2.02)
3.30 (2.41,4.19)	− 0.52 ( − 1.81,0.76)	− 0.49 ( − 1.64,0.66)	− 1.30 ( − 4.90,2.30)	− 0.33 ( − 2.02,1.36)	Sling exercise

Posterior means 95% Bayesian credible intervals are calculated by column–row under the fixed effects model assuming consistency.Mean difference < 0 favors the intervention in the column; mean difference > 0 favors the intervention in the row

### Ranking probability

**Fig. 4:**
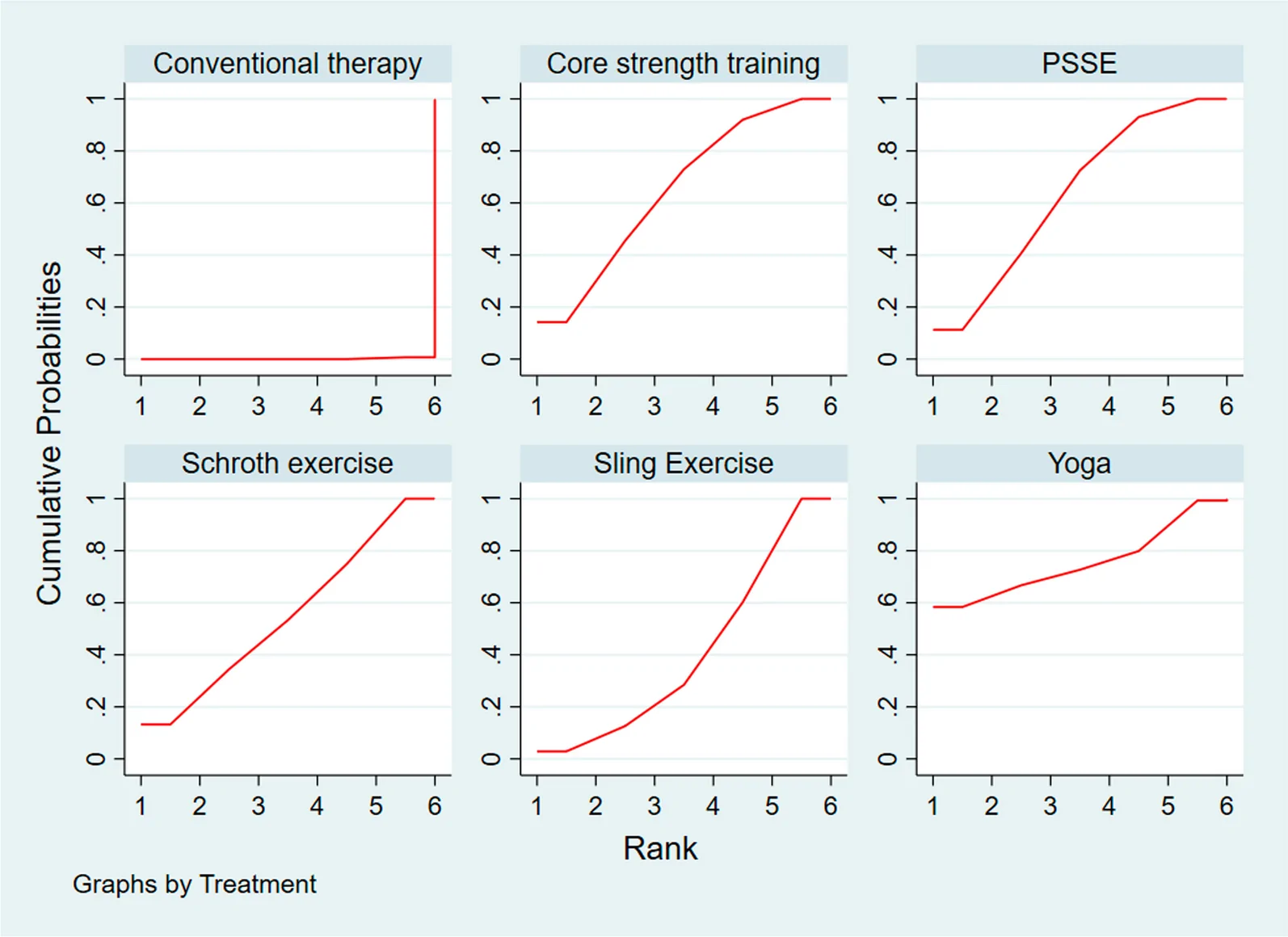
SUCRA curves of the effectiveness of each exercise form. The larger the area under the SUCRA curve, the greater the cumulative probability of ranking high for that intervention

Network meta-analysis supported ranking the various interventions. From the results of the network meta-analysis, a ranking of the effects of each intervention could be calculated. However, we recommend using cumulative probability to rank interventions rather than ranking them according to the best ranking probability, because ranking the best probability does not take into account the uncertainty of relative effect estimation and relative ranking. Figure 4 shows the cumulative probability curve of the ranking of various interventions, and the larger the area under the curve, the greater the cumulative probability of the ranking of the intervention. Table 3 shows the SUCRA value for each intervention, which represents the probability that an intervention is one of the best options, with a SUCRA value of 100% indicating that the intervention is most effective and a SUCRA value of 0% indicating that the intervention is least effective. After analysis, it was found that among all interventions, yoga (SUCRA value = 76.0) had the largest area under the curve, followed by core strength training (SUCRA = 64.7), PSSE (SUCRA = 63.8), Schroth exercise (SUCRA = 55.3), and sling exercise (SUCRA = 40.2). The intervention with the smallest area under the curve was conventional therapy (SUCRA = 0.1). Therefore, it can be concluded that among all interventions, yoga has the most obvious effect on reducing Cobb angle, followed by core strength training, PSSE, Schroth exercise, sling exercise, and conventional therapy.

**Table 3:** SUCRA values, optimal probability, and mean rank of different interventions

Intervention	SUCRA	PrBest/%	MeanRank
Conventional therapy	0.1	0.0	6.0
Core strength training	64.7	13.7	2.8
PSSE	63.8	11.2	2.8
Yoga	76.0	59.1	2.2
Schroth exercise	55.3	13.4	3.2
Sling exercise	40.2	2.7	4.0

## Discussion

Due to the complexity of the pathogenesis of AIS, it is not yet possible to determine precisely the mechanism of the patient's pathogenesis in clinical practice. However, abnormal gene expression, abnormal biochemical indicators, neurological abnormalities, and muscle strength imbalances are the four major factors recognized by the medical community as triggering AIS. Among the studies on abnormal gene expression, one researcher found that there may be familial aggregation of AIS; Ogilvie collected data on the family histories of 145 AIS patients and created a database that was compared to determine associations with other AIS families, and the results showed that there was up to a 97% correlation between AIS families, which means that at least one of the main genes was inherited [44]; Fan Hengwei et al. explored the pathogenesis of AIS by applying gene microarray technology and found that the gene expression of BM-MSCs from AIS patients was abnormal during the process of lipidogenic differentiation [45]; it has also been found that the rearrangement phenomenon of the 16p11.2 gene [46] and the locus on chromosome 10q24.31 [47] are associated with AIS. In studies related to abnormal biochemical markers; researchers have found that abnormal bone metabolism [48], disrupted growth hormone secretion [49], melatonin deficiency [50], and abnormal leptin activity [51] can induce AIS. In the study of neurological abnormalities, abnormal somatosensory evoked potentials (SEP) are one of the main focuses of researchers' attention; SEP is an important factor affecting the body's posture and gait balance. Cheng [52] analyzed the posterior tibial nerve evoked potentials and whole spine MRI examinations of healthy individuals and patients with AIS, and it was concluded that the sensory dysfunction of the torso may be one of the most important causative factors leading to AIS. A healthy spine is in equilibrium mechanically and physiologically, but when paraspinal muscle strength is uneven, muscle fibers are mutated, etc., which affects the spine's force loading in three-dimensional space for a prolonged period of time; the probability of AIS is dramatically increased [53].

In summary, after categorizing the triggering factors of AIS, it provides the possibility to explore the mechanism of exercise intervention to improve AIS. The human body has a strong adaptive capacity, and the brain, as the command center of the human body, also has plasticity. Through exercise, the cells and synapses of the human nervous system can undergo physiological adaptive changes, resulting in new neural connections and functional changes [54]. RUF [55] has shown that AIS is induced by abnormalities in the functioning of the brainstem and spinal cord, which make the patient's proprioceptive functions and body posture control dysfunctional. Moreover, exercise is effective in improving the strength of the muscles around the spine, thereby restoring them to a physiological and mechanical state of balance, which is one of the reasons why exercise therapy has attracted the attention of researchers [56]. Meanwhile, some researchers have also conducted meta-analysis on the therapeutic efficacy of exercise interventions for scoliosis [57, 58], and the results have all shown that exercise interventions can effectively reduce the degree of scoliosis in patients. However, current related studies lack a visual comparison of the therapeutic effects between individual exercise interventions; therefore, this study compared individual exercise interventions through meta-analysis to determine which of them is more effective in improving AIS.

Through systematic review and network meta-analysis, it was found that exercise therapy was more effective in improving AIS than conventional therapy. Therefore, eligible AIS patients can participate in core strength training, yoga, sling exercise, etc., under the guidance of a professional for faster recovery. Among all exercise therapies, the use of yoga intervention may have the best effect on AIS improvement. Compared with conventional therapy, yoga reduced the average Cobb angle by 4.60 degrees and ranked first in the cumulative probability of improving the effect of AIS among all exercise interventions. The other interventions ranked in order of effectiveness were core strength training, PSSE, Schroth exercise, and sling exercise.

According to the final results of the intervention effects of various exercise interventions on AIS, the intervention effects of the Schroth exercise and sling exercise ranked the last two. However, this result should be interpreted with caution, because among all the literature included in this study, there was only one article using these two types of exercise as intervention measures, and the sample size of the experimental group in the Schroth exercise intervention experiment was only 17, while the intervention duration of the RCT experiment using sling exercise intervention was only 4 weeks. Therefore, before determining the intervention effect of the Schroth exercise and sling exercise on AIS, more relevant studies should be introduced, and the sample size and duration of experimental intervention should be increased. The purpose of this study is to compare the improvement effects of different exercise measures on AIS, while due to space limitations, this paper only explores the general mechanisms by which exercise interventions can improve the pathologies of AIS patients. However, the specific mechanisms by which each exercise intervention improves the conditions of AIS patients are not discussed in detail in this paper. Finally, the results should be interpreted and adopted with caution due to significant publication bias in the included studies. 

## Data Availability

The datasets used and/or analyzed during the current study are available from the corresponding author on reasonable request.
